# Lamotrigine compromises the fidelity of initiator tRNA recruitment to the ribosomal P-site by IF2 and the RbfA release from 30S ribosomes in *Escherichia coli*

**DOI:** 10.1080/15476286.2023.2253395

**Published:** 2023-09-07

**Authors:** Sudhir Singh, Kuldeep Lahry, Chandra Sekhar Mandava, Jitendra Singh, Riyaz Ahmad Shah, Suparna Sanyal, Umesh Varshney

**Affiliations:** a Department of Microbiology and Cell Biology, Indian Institute of Science, Bangalore, India; b Department of Cell and Molecular Biology, Biomedical Centre, Uppsala University, Uppsala, Sweden; c Jawaharlal Nehru Centre for Advanced Scientific Research, Bangalore, India

**Keywords:** Ribosome, lamotrigine, Ltg, initiation factor 2, initiator tRNA

## Abstract

Lamotrigine (Ltg), an anticonvulsant drug, targets initiation factor 2 (IF2), compromises ribosome biogenesis and causes toxicity to *Escherichia coli*. However, our understanding of Ltg toxicity in *E. coli* remains unclear. While our *in vitro* assays reveal no effects of Ltg on the ribosome-dependent GTPase activity of IF2 or its role in initiation as measured by dipeptide formation in a fast kinetics assay, the *in vivo* experiments show that Ltg causes accumulation of the 17S precursor of 16S rRNA and leads to a decrease in polysome levels in *E. coli*. IF2 overexpression in *E. coli* increases Ltg toxicity. However, the overexpression of initiator tRNA (i-tRNA) protects it from the Ltg toxicity. The depletion of i-tRNA or overexpression of its 3GC mutant (lacking the characteristic 3GC base pairs in anticodon stem) enhances Ltg toxicity, and this enhancement in toxicity is synthetic with IF2 overexpression. The Ltg treatment itself causes a detectable increase in IF2 levels in *E. coli* and allows initiation with an elongator tRNA, suggesting compromise in the fidelity/specificity of IF2 function. Also, Ltg causes increased accumulation of ribosome-binding factor A (RbfA) on 30S ribosomal subunit. Based on our genetic and biochemical investigations, we show that Ltg compromises the function of i-tRNA/IF2 complex in ribosome maturation.

## Introduction

Bacterial ribosomes consist of a small subunit (30S), comprising 16S rRNA and about 21 ribosomal proteins, and a large subunit (50S), comprising one molecule each of 23S and 5S ribosomal RNAs (rRNAs) and about 33 ribosomal proteins [1]. The endonucleolytic cleavages of a precursor RNA even as it is being transcribed, followed by further processing of the cleaved products at the 5’ and 3’ ends by a number of RNases generate mature 16S, 23S and 5S rRNAs from their immediate precursors [2]. An accurate biogenesis of the two subunits, the ribonucleoprotein particles, requires assembly factors that serve as GTPases, RNA chaperons, RNA and protein modifying enzymes and RNases [3]. While the correct assembly of ribosomes has been achieved in vitro [4,5], the efficient assembly/biogenesis under physiological conditions requires distinct assembly factors. About 60 such factors are used in prokaryotes [6].

During maturation of the 30S subunit, ribosome-binding factor A (RbfA), one of the early biogenesis factors, binds near the 3’ end of 16S rRNA [7]. Subsequently, RbfA is replaced by ribosomal small subunit dependent GTPase (RsgA), a late stage 30S dependent GTPase. RsgA binds near the decoding centre of the 30S and prevents premature binding of the initiation factors (IF1, IF2 and IF3), A-site and P-site tRNAs and the 50S [8]. The deficiency of RsgA can be compensated for by mutations in RbfA [9]. Recent studies have shown the involvement of IF3 [10] and ribosomal large subunit pseudouridine synthase D (RluD) [11] in the release of RbfA from 30S in *E. coli*. Any deficiencies in ribosome biogenesis may result in the loss of fidelity of translation, and the aberrantly produced ribosomes may be toxic/detrimental to the cell [12]. The ribosome biogenesis defects may also alter drug resistance and virulence of the bacteria [13,14]. Further, the ribosome assembly defects in humans give rise to various ribosomopathies [15,16]. Thus, a better understanding of ribosome biogenesis is an important area of research.

Stokes and co-workers, screened for compounds that may affect ribosome biogenesis and showed that a small molecule, lamotrigine (Ltg), used as an anticonvulsant, caused ribosome biogenesis defect in *E. coli,* especially at a low temperature of ~ 15°C [17]. Isolation of Ltg resistant (Ltg^R^) strains identified IF2 as its target. In *E. coli*, *infB* gene codes for two major isoforms of IF2 (IF2α of 97.3 kDa, and IF2β of 79.7 kDa). The shorter form (IF2β) is a mixture of two sub-isoforms (IF2β_1_ and IF2β_2_) arising from in-frame downstream initiation codons at positions 158 and 165, respectively [18]. The IF2α (890 amino acids) comprises three major domains viz. the N -terminal domain, the middle G domain and the C- terminal domain. The functions of the middle G and C- terminal domains were shown to be in nucleotide binding (GTP/GDP/ppGpp) and in i-tRNA binding, respectively [19]. The *N*- terminal of the full-length IF2 comprises three consecutive subdomains, I (~157 amino acids), II (~140 amino acids) and III (~100 amino acids). The IF2β isoforms lack domain I of the full-length IF2. Ltg targets domain II of IF2 in the N-terminal region [17].

Earlier, we reported that i-tRNA helps in biogenesis of the ribosome by assisting at the ultimate steps of the maturation of the 5’ and 3’ ends of 16S rRNA [20]. Depletion of i-tRNA or overexpression of an i-tRNA having mutations in its characteristic three consecutive GC pairs (3GC pairs) in the anticodon stem, conferred cold sensitivity to *E. coli* and led to the accumulation of immature forms of 16S (17S) rRNA [20]. Since i-tRNA interacts with IF2 [21], we investigated if there is a connection between the Ltg mediated ribosome biogenesis defect and the role of IF2 with i-tRNA. We show that Ltg results in increased levels of IF2 in cells, allows initiation with elongator tRNA and increases accumulation of RbfA on the 30S. The overexpression of i-tRNA rescues *E. coli* from the Ltg toxicity. These observations strengthen our earlier findings on the role of the i-tRNA in ribosome maturation, highlight the importance of the optimal abundance of i-tRNA and IF2 and elaborate on the mechanism of Ltg action on ribosome and in *E. coli*.

## Materials and methods

### Bacterial strains, growth conditions, plasmid, and DNA oligomers

Bacterial strains, plasmid and DNA oligomers used in the study are listed in Tables 1 and 2. *E. coli* KL16 (or KL16), its derivatives and other strains were grown in LB broth. Bacto agar (1.5%) was included for growth on solid media. Unless mentioned otherwise, media were supplemented with ampicillin (Amp, 100 µg/ml), chloramphenicol (Cm, 30 µg/ml), kanamycin (Kan, 25 µg/ml) or tetracycline (Tet, 7.5 µg/ml). Ltg (CAYMAN, USA) stock (50 mM) was prepared by dissolving it in DMSO and stored at −20°C.

**Table 1.** List of strains/plasmids used.

Strain/Plasmid	Description	Reference
*E. coli* KL16	*E. coli* K-12 *λ*^−^ *e14- relA1 spoT1 thiE1*	[22]
*E. coli* TG1	*supE hsd*∆5 *thi ∆*(*lac-proAB*) F` [*traD36 proAB* + *lacIq lacZ∆M15*]	[23]
*E. coli* BL21 (Rosetta ^TM^)	F^–^ *ompT gal dcm lon hsdS*_*B*_(r_B_^–^ m_B_^–^) [*malB*^+^]_K-12_(λ^S^)	[24]
*ΔmetZWV*	Deletion of 3 copies of i-tRNA genes (*metZ*, *metW* and *metY*) by kanamycin cassette insertion followed by removal of Kan cassette using pCP20 plasmid.	This Study
pACDH-empty	A low copy number plasmid with ACYC ori of replication, compatible with ColE1 origin of replication and harbouring Tet^R^	[25]
pACDH-*infB*	*E. coli infB* gene cloned in pACDH plasmid	[26]
pACDH-*infB*_Δ17_	*E. coli infB* gene with deletion of 17 amino acid in domain II cloned in pACDH plasmid	This study
pACDH-*metY*	*E. coli metY* gene cloned in pACDH plasmid	[27]
pEmpty	A high copy number plasmid with ColE1 origin of replication and Amp^R^	[28]
p*metY*	pCAT_am1_*metY* derivative harbouring wild type *metY* gene but lacking the CAT_am1_ reporter gene (Amp^R^).	[29]
pCAT_am1_	Renamed from pRSVCATam1.2.5. harbours a CAT reporter gene with UAG initiation codon (Amp^R^)	[28]
p*metY*_3GC/CUA_	pCAT_am1_ *metY*_CUA_ derivative harbouring the *metY*_CUA_ gene with 3GC (U29:A41/C30:G40/A31:U39) mutation but lacking the CAT_am1_ reporter gene	[29]
pHIS-IF2	*E. coli infB* gene with N terminal 6X His tag cloned in pACDH plasmid	[30]
pHIS-IF2_Δ17_	*E. coli infB* gene with N terminal 6X His tag and deletion of 17 amino acid in domain II of IF2 cloned into pACDH plasmid	This Study
p*metY*_3GC_	Initiator tRNA cloned in to high copy number plasmid having 3GC mutation (U29:A41/C30:G40/A31:U39) but WT anticodon (CAU).	[28]
pKD4	Amp^R^ and Kan^R^, Kan^R^marker is flanked by FRT sequences.	[31]
pKD46	Amp^R^, harbours λ Red recombination genes (γ, β and exo)	[31]
pCP20	Amp^R^, yeast Flp recombinase gene, FLP, and Cm^R^	[31]
pSUB11	Amp^R^ and Kan^R^, Kan^R^ used to append 3X-FLAG tag sequence at the 3'-end of the gene ORF	[32]

**Table 2.** List of DNA oligomers used in the study.

S.N.	Sequence 5’ to 3’
*rbfA_*FLAG Fp	GAACGTCGTGTTAACCCGGACGACAGCAAGGAGGACGA CTACAAAGACCATGACGG
*rbfA_*FLAG Rp	CAAAACGCCGTTAATGTCGCGACCGCGACGACGAGGCA TTAGTCCTCCTCATATGAATATCCTCCTTAG
*infB*_His-Fp	AACCCATGGGCCATCATCATCATCATCATAGCAGCATGACAGATGT
*infB*_Rp	AAAAAGCTTAAGCAATGGTACG
IF2_Δ17_ Fp	CGTAAACTCGAAGAAGAAG
IF2_Δ17_ Rp	GCGGGCTTTTTCAGCCTGG
16S	TCTTCGCGTTGCATCGAATT
p16S–3’	TGTGTGAGCACTGCAAAGAACGCTTTAAGG

### Dilution spotting plate assays and growth curve analyses

To check the growth of various *E. coli* strains, dilution spotting plate assays were done. Isolated colonies from freshly streaked plates were inoculated into 2 ml LB to prepare overnight cultures, which were then diluted 100-fold into fresh LB and grown till mid log phase (~0.5 to 0.6 OD_600_) at 37°C, and after equalizing to 1 OD_600_ by pelleting the required volume from different backgrounds, the cultures were serially diluted (10^−1^, 10^−2^, 10^−3^ and 10^−4^-fold) in LB, and 200 µl aliquots of the diluted cultures were taken into 96-well ELISA plate. A spotter dipped into the diluted cultures was gently touched on to LB agar plate containing various antibiotics or Ltg (as required) and incubated at specified temperatures for specified hours. For growth curve analysis, overnight grown cultures were serially diluted up to 1000-fold in fresh LB, and 200 µl of diluted cultures were taken into the honeycomb well plates in triplicates to carry out growth analysis in Bioscreen C (Labsystem, Finland). Growth was recorded at 1 h intervals for up to 24 h by measuring O.D. at 600_nm_.

### Northern blotting

To check the level of immature 16S rRNA, cultures were grown till mid log phase and total RNA was isolated with hot phenol. Briefly, cells from 2 ml culture were pelleted and resuspended into 450 µl lysis buffer (30 mM Tris-HCl, pH 8.0, 0.1 M NaCl, 5 mM Na_2_EDTA, 1% SDS and 8 mM β-mercaptoethanol) and subjected to RNA extraction. The RNA was separated on 1.2% agarose gel, electroblotted onto Nytran membrane at 6 V for 2 h 30 min, fixed onto the membrane using UV crosslinker at 120 mJ/cm^2^ (CL 1000-UV products) and probed sequentially with ^32^P end labelled DNA oligomers against 16S–3’ immature rRNA end and mature region of 16S rRNA (Table 2). The blot was exposed to phosphor-imager screen and quantified.

### Isolation of strains resistant to lamotrigine and characterization of infB gene

To isolate Ltg^R^ *E. coli* KL16, overnight grown culture was diluted 500-fold in 2 ml LB containing 200 µM Ltg and incubated at 15°C for 2 weeks (turbidity appeared on 11^th^ day). The cells were sub-cultured and grown again in 200 µM Ltg at 15°C. The growth appeared within 3 days. The culture was streaked on LB agar and incubated at 37°C. Six isolated colonies were inoculated in 2 ml LB, sub-cultured for three successive generations at 37°C and streaked on LB agar plate. The isolated colonies were checked for growth on LB-agar containing Ltg (20 and 40 µM) at 22°C (Fig. S1A). Control plates contained equivalent concentration of DMSO carrier. Colonies from the Ltg^R^ strain but not the control strain grew on the Ltg plates at 22°C. The *infB* alleles from the cultures were PCR amplified and sent for DNA sequencing (Fig. S1B). The *infB* allele having a deletion in the domain II from the strain named isolate 3 (with largest deletion) was used in further studies.

### Generation of isogenic strains of KL16 having IF2_WT_ and IF2_Δ17_

The *kan* cassette from pSUB11 was PCR amplified using *rbfA*-Fp and *rbfA*-Rp primers (Table 2) for insertion in the downstream region of *rbfA* (*infB* locus) in KL16 and its Ltg^R^ isolate 3 (harbouring pKD46) by electroporation. Transformants were selected on Kan plates, confirmed for *kan* marker insertion downstream to *rbfA* by PCR and used to raise P1 phage lysates (Fig. S2). P1 phage mediated transductions were used to generate isogenic strains of KL16 or TG1 harbouring *infB* alleles (IF2_WT_ and IF2_Δ17_) (Fig. S3).

### Cloning of IF2_Δ17_ into pACDH

IF2_Δ17_ was generated by site-directed mutagenesis of the plasmid clone of IF2_WT_ as template using inverse PCR method (IF2_Δ17_ Fp/Rp, Table 2) by initial denaturation at 94°C for 3 min, followed by incubations at 94°C for 1 min, 55°C for 1 min, 70°C for 6 min for 16 cycles and then 70°C for 7 min. Reactions were treated with DpnI to degrade the original template, ligated using T4 DNA ligase and transformed into TG1. Transformants were used to isolate the recombinant plasmid and confirmed by DNA sequencing.

### Purification of IF2

Plasmid pHis-IF2 having (His)_6_ tag at N-terminal [30] was used to purify IF2_WT_. For purification of IF2_Δ17_, pHis-IF2_Δ17_ was generated by cloning of NcoI and HindIII digested PCR amplicon (infB-His-Fp/infB-Rp, Table 2) from genomic DNA of isolate 3 into similarly digested pHis-IF2. The expression plasmids were confirmed by sequencing and introduced into *E. coli* BL21 (Rosetta^TM^), and cultures (3 L LB) of freshly obtained transformants were grown at 37°C till mid-log phase, induced with 0.1 mM IPTG, grown further for 5 h and harvested, and the pellets were resuspended in 30 ml ice-cold buffer A [20 mM Tris-HCl, pH 7.5, 5% (v/v) glycerol, 500 mM NaCl, 2 mM β-mercaptoethanol and 10 mM imidazole] with 1 mM phenylmethylsulfonyl fluoride (PMSF). The cells were lysed by sonication and centrifuged at 100,000 g at 4°C for 1 h. The supernatant was loaded onto a pre-equilibrated Ni-NTA column (HisTrap HP, 5 ml, GE Healthcare, USA), followed by washing and elution of proteins with a gradient (10 mM to 1 M) of imidazole in 30 ml. The eluted fractions were analysed by SDS-PAGE. The fractions containing pure proteins were pooled and concentrated by centrifugal filters (50 kDa cut off, Merck) to 500 µl, loaded onto gel filtration column (Superdex 200, GE Healthcare, USA) and eluted in a buffer containing (20 mM Tris-HCl pH 7.5, 10% glycerol, 2 mM β-mercaptoethanol, and 500 mM NaCl). Pooled fractions were dialysed against 20 mM Tris-HCl, pH 7.5, 2 mM β-mercaptoethanol, 100 mM NaCl and 50% (v/v) glycerol and stored at −20°C.

### GTPase activity assays

To determine ribosome-dependent GTPase activity of IF2 proteins, 70S ribosomes [33] (0.865 OD_260_, activated at 37°C for 15 min) were taken in 15 μl reactions containing 4.7 μg IF2_WT_ or IF2_Δ17_ (3.22 μM) in 50 mM Tris-HCl (pH 7.5), 50 mM NH_4_Cl, 10 mM MgCl_2_, 7 mM β-mercaptoethanol and 266 μM GTP with trace amounts (0.2 μCi) of [α- ^32^P] GTP, incubated at 28°C for 35 min and stopped by addition of 15 μl of 40% formic acid. Aliquots (1.5 μl) from the reactions were spotted on PEI-Cellulose TLC plates (Polygram 300 CEL PEI-/UV254, Aldrich) and developed using 1.5 M KH_2_PO_4_, pH 3.5, as mobile phase [34]. The spots corresponding to GTP and GDP were quantified by a BioImage analyser (FLA2000, Fuji Film, Japan), and the pmoles of GDP formed were calculated as [P/(S+P)]*400. The [α- ^32^P] GTP treated with *E. coli* nucleoside diphosphate kinase [35] was used to generate [α- ^32^P] GDP marker. IF2 (WT or Δ17) at 4.7 μg each were incubated with either Ltg (10, 100, 200 or 300 μM) or equal volumes of water (untreated) on ice for 15 min before adding to the reaction. For time kinetics, Ltg (53 μM) was used and IF2 proteins were incubated for 45 min on ice. The reactions were stopped with 15 μl of 40% formic acid at 0, 5, 10, 20 and 40 min.

### Dipeptide formation assay

All the translation factors and ribosomes for *in vitro* translations were prepared as earlier [36]. Dipeptide formation experiments were conducted at 37°C in 1X HEPES-polymix buffer containing 5 mM HEPES (pH 7.5), 100 mM KCl, 5 mM NH_4_Cl, 5 mM Mg(OAc)_2_, 0.5 mM CaCl_2_, 1 mM spermidine, 8 mM putrescine and 1 mM 1,4-dithioerythritol and energy pump components 1 mM GTP, 1 mM ATP, 10 mM PEP, pyruvate kinase (50 μg/ml) and myokinase (2 μg/ml), unless mentioned otherwise. A ‘ribosome mixture’ containing 70S ribosomes (1 μM), f [3]H]-Met-tRNA^fMet^ (2 μM), XR7 MFL mRNA (2 μM), AACAAU*UAAGGAGG*UAUUAA**AUGUUCCUG**UAAGAAU (Shine-Dalgarno sequence is shown in italics and the coding region is shown in bold), IF1, IF3, and IF2 (1 μM each) was incubated for 10 min at 37°C to allow for the formation of initiated ribosomes. Similarly, a ‘ternary-complex mixture’ containing tRNA^Phe^ enriched bulk tRNA (10 μM), EF-Tu (10 μM), EF-Ts (2 μM), PheRS (0.2 units/μl) and Phe (200 μM) was incubated for 15 min at 37°C to form EF-Tu·GTP·Phe-tRNA^Phe^ ternary complex. The two mixtures were rapidly mixed in equal volumes in a temperature-controlled (37°C) quench-flow instrument (RQF-3, KinTek Corp.), and the reactions were stopped at different times by rapid addition of 17% formic acid (final concentration) as a quencher. The dipeptide formation was analysed by RP-HPLC [37]. For the dipeptide formation experiments starting from the subunits, 50S (1 μM) was supplied in the ‘ternary-complex mixture’, while the ‘ribosome mixture’ contained purified 30S subunits (1 μM) [38]. In both the assays, 400 μM Ltg was added in the ‘ribosome mixture’ for a final concentration of 200 µM Ltg in the dipeptide reaction.

### Polysome profiling

Cell lysates were prepared [39] from freshly grown cultures at 28°C to OD_600_ of ~ 0.5, following inoculations with 1% overnight cultures in 100 ml LB with or without Ltg. Approximately, 12 OD_260_ of the lysates prepared in buffer A (20 mM Tris-Cl, pH 8.0, 60 mM NH_4_Cl, 100 mM MgCl_2_ and 4 mM β-mercaptoethanol) containing 5% sucrose were loaded onto 10 to 35% sucrose gradient and centrifuged at 36,000 rpm for 4 h in ultracentrifuge (Beckman, USA) using SW41 rotor. Fractions were collected using the Biocomp machine piston gradient fractionator.

### Immunoblotting

To check the cytoplasmic level of IF2, equal ODs of the cultures were harvested at mid-log phase, resuspended into 100 µl of 1X SDS dye, boiled at 95°C for 7 min, vortexed and centrifuged at 13,000 rpm for 2 min. Samples corresponding to 0.2 OD_600_ of culture were separated on 12% SDS PAGE and transferred on to polyvinylidene difluoride (PVDF) membrane at 15 V for 1 h 30 min. The membrane was blocked in 1X-TBST [20 mM Tris-HCl, pH 7.5, 0.9% NaCl and tween-20 (0.2% V/V)] containing 5% skimmed milk powder, followed by incubation with anti-IF2 rabbit antibodies (1:6000 dilution) at 4°C overnight under shaking conditions. The membrane was washed thrice with 1X-TBST for 15 min each, and then incubated in IgG-HRP, anti-rabbit antibody (1:5000 dilution) for 2 h, washed again thrice with 1X-TBST for 15 min each, developed with Lumina Fort HRP substrate and scanned using Chem Doc (GE, USA). To check the level of FLAG_tagged RbfA in the various fractions of polysomes, total proteins in the collected fractions were precipitated with 2 volumes of chilled acetone by keeping at −20°C overnight. Next day, the tubes were centrifuged at 13,000 rpm for 5 min, and the obtained pellets were air dried and taken up in to 20 µl of 1X SDS dye, boiled at 95°C for 7 min, vortexed and centrifuged at 13,000 rpm for 2 min. Total proteins were separated on 12% SDS-PAGE and transferred onto PVDF membrane at 10 V for 1 h. To check the levels of RbfA (FLAG tagged), the primary antibody (1:10,000 dilution) against FLAG and secondary antibody anti-mouse antibody (1:5000 dilution) were used [11].

## Results

### Lamotrigine toxicity in E. coli increases upon overexpression of IF2

**Figure 1.**
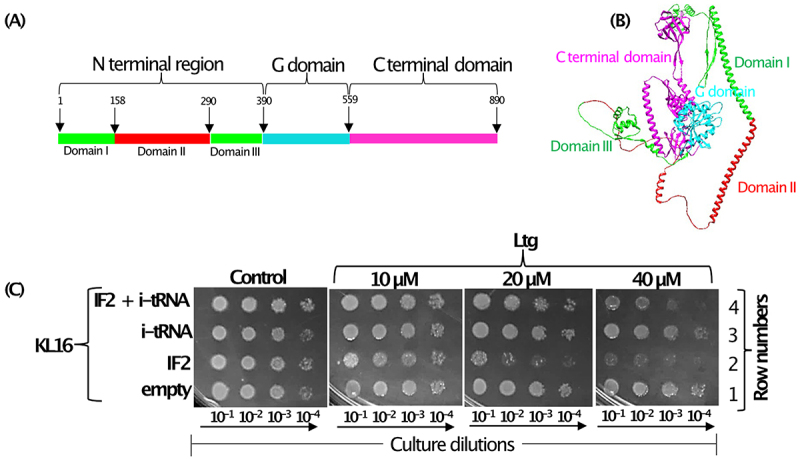
(A) Schematic representation of different domains of IF2 having N terminal region (green colour) encompassing domain II (highlighted in red colour), G domain (cyan colour) and C terminal domain (magenta colour). (B) Structure of the full-length IF2 downloaded from Alpha fold having same colour code for different domain as in (Figure 1A). (C) Dilution spotting plate assay showing growth of serially diluted (10^−1^, 10^−2^, 10^−3^ and 10^−4^) log phase cultures of KL16 strains harbouring empty plasmid pACDH (Tet^R^) and pEmpty (Amp^R^) plasmid borne genes of IF2, i-tRNA alone or together (IF2 and i-tRNA) at 28°C for 36 h in the presence of increasing amounts of Ltg. pACDH having ACYC origin of replication expressing IF2 and pEmpty having ColE1 origin of replication expressing *metY* (i-tRNA) are compatible.

We began our investigations based on an earlier observation [17] that Ltg targets IF2 and that Ltg resistance arises by the mutations in the subdomain II in the N-terminal region of IF2 (Figure 1A). As IF2 binds i-tRNA to locate it to P-site during the step of initiation, to understand the mechanism of Ltg mediated toxicity, we overexpressed IF2 and i-tRNA individually or together (IF2 and i-tRNA) in *E. coli* KL16 and checked the strain growth in the presence of increasing concentrations of Ltg at 28°C. Plate dilution spotting assay shows that Ltg mediated growth inhibition of KL16 was enhanced upon overexpression of IF2 (Figure 1C, compare row 2 with 1). However, the overexpression of i-tRNA protected the strain from growth inhibition by Ltg in the presence of overexpressed IF2 (Figure 1C, compare rows 2 and 4). When compared with the empty vector, overexpression of i-tRNA alone did not show a significant effect on the growth rescue of KL16 on the plates (Figure 1C, compare rows 1 and 3). Although, growth recovery of Ltg treated KL16 upon overexpression of i-tRNA was visible in LB broth (Fig. S4). Overall, the plate dilution spotting assay shows that Ltg causes toxicity to *E. coli*; the toxicity is elevated by the presence of excess IF2 but rescued by the simultaneous overexpression of i-tRNA.

### The presence of the 3GC mutant i-tRNA or depletion of cellular i-tRNA enhance lamotrigine toxicity

**Figure 2.**
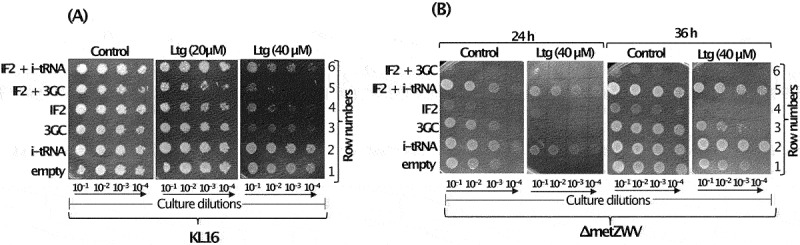
(A) Dilution spotting plate assay showing growth of serially diluted culture (10^−1^, 10^−2^, 10^−3^ and 10^−4^) of KL16 harbouring plasmid borne copies of genes of IF2, i-tRNA, 3GC mutant i-tRNA mutant, i-tRNA with IF2 and, 3GC mutant i-tRNA with IF2 at 28°C in the presence of Ltg (20 and 40 µM) for 36 h. Plasmid, pACDH (ACYC ori) was used for overexpression of IF2 and pEmpty (ColE1 ori) was used for overexpression of i-tRNA or the 3GC mutant i-tRNA. (B) Dilution spotting plate assay of the serially diluted culture (10^−1^, 10^−2^, 10^−3^ and 10^−4^) of KL16*ΔmetZWV* having overexpressed IF2, i-tRNA, 3GC i-tRNA mutant, and i-tRNA with IF2 and 3GC i-tRNA mutant with IF2 at 28°C in the presence of Ltg (40 µM) for 24 h and 36 h. Same set of plasmid constructs were used as in (Figure 2A).

Earlier, we showed that the highly conserved feature of the three consecutive GC base pairs (3GC or GC/GC/GC) in i-tRNA anticodon stem (G29:C41; G30:C40; G31:C39) facilitates ribosome maturation [20]. When the 3GC base pairs, GC/GC/GC, were mutated to UA/CG/AU, the resulting ‘3GC mutant’ i-tRNA was inactive in initiation and caused ribosome biogenesis defect [20]. As i-tRNA helped in recovery from the Ltg-mediated toxicity (Figure 1), to further our understanding of the role of i-tRNA-mediated rescue, we used the 3GC mutant i-tRNA. Overexpression of the mutant i-tRNA enhanced Ltg toxicity in KL16 (Figure 2A, compare rows 3 and 1). The Ltg-mediated toxicity was enhanced upon simultaneous overexpression of IF2 (Figure 2A, row 5). As expected, overexpression of wild type i-tRNA along with IF2 (Figure 2A, compare rows 4 and 6) rescued the strain from Ltg toxicity.

To further validate the role of i-tRNA, we used the *ΔmetZWV* strain, where three (*metZWV*) of the four i-tRNA genes are deleted and the strain survives on the remaining single i-tRNA gene (*metY*). In this experiment (Figure 2B), we observed a more severe growth defect upon overexpression of the 3GC i-tRNA mutant alone or in combination with IF2 in the presence of Ltg (Figure 2B, rows 3 and 6, 24 h and 36 h sets). In the *ΔmetZWV* background, only combinations with overexpressed wild type i-tRNA survived better in the presence of Ltg (Figure 2B, rows 2 and 5, 24 h and 36 h sets). The data in Figure 2A,B show that Ltg toxicity increases with a stoichiometric excess of IF2 over i-tRNA or when i-tRNA mutant deficient in initiation is used.

### Effect of the lamotrigine through domain II of IF2

**Figure 3.**
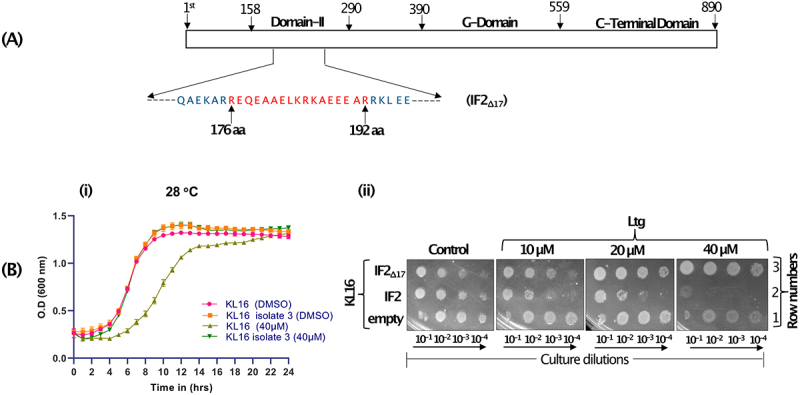
(A) Schematic showing different domains of IF2 (with domain II enlarged between 158^th^ to 290^th^ amino acid, and the region deleted in IF2_Δ17_ is shown red). (B) Growth curves showing effect of Ltg in KL16 having IF2_WT_ and IF2_Δ17_. The line joins the mean of three replicates, and error bars represent the standard deviation. (C) Dilution spotting plate assay showing growth of serially diluted culture (10^−1^, 10^−2^, 10^−3^ and 10^−4^) of KL16 having overexpressed IF2_WT_ or IF2_Δ17_ (in pACDH) at 28°C in the presence of Ltg (10, 20 and 40 µM) for 36 h.

The earlier study [17] demonstrated that a mutant IF2 (IF2_Δ8_) with a deletion of 8 amino acid in domain II conferred Ltg resistance (Ltg^R^) in *E. coli*. To understand the effect of Ltg on KL16, we repeated the process of isolation of Ltg^R^ *E. coli* KL16 (differing from the strain used before), and PCR amplified *infB* from the Ltg^R^ isolates. Plate assays showed that all six isolates grew well in 20 µM and 40 µM Ltg while the control strain was unable to grow (Fig. S1A). Analysis of IF2 showed the deletion of a few to several amino acids in domain II (Fig. S1B). We then worked with isolate 3 (IF2_Δ17_, Figure 3A) having the largest deletion of 17 amino acids (176^th^ to 192^nd^ position) in domain II. To confirm that the Ltg^R^ phenotype was indeed due to IF2_Δ17_, we transferred the mutant *infB* allele from isolate 3 to fresh KL16 background by P1 phage-mediated transduction. Analysis of the transductants at 28°C showed that while the growth of the strain having wild type IF2 (IF2_WT_) was susceptible to Ltg, that with IF2_Δ17_ was not (Figure 3B, i). These data show that Ltg-mediated effects were mediated through IF2 domain II. The *infB* allele of isolate 3 (IF2_Δ17_) was also cloned in pACDH. In the dilution spotting plate assay, increasing Ltg showed toxicity to KL16 (Figure 3B, ii, row 1, compare control panel with the Ltg panels). The toxicity increased (Figure 3B, ii, row 2) upon overexpression of IF2_WT_. However, when IF2_Δ17_ was overexpressed, Ltg treatment did not result in growth defects even in its increasing concentrations (Figure 3B, ii, row 3), suggesting once again (in a different strain and a different size deletion in *infB*) that Ltg toxicity is mediated through IF2 domain II.

### Lamotrigine increases cytosolic IF2_WT_ levels impacting ribosome biogenesis

**Figure 4.**
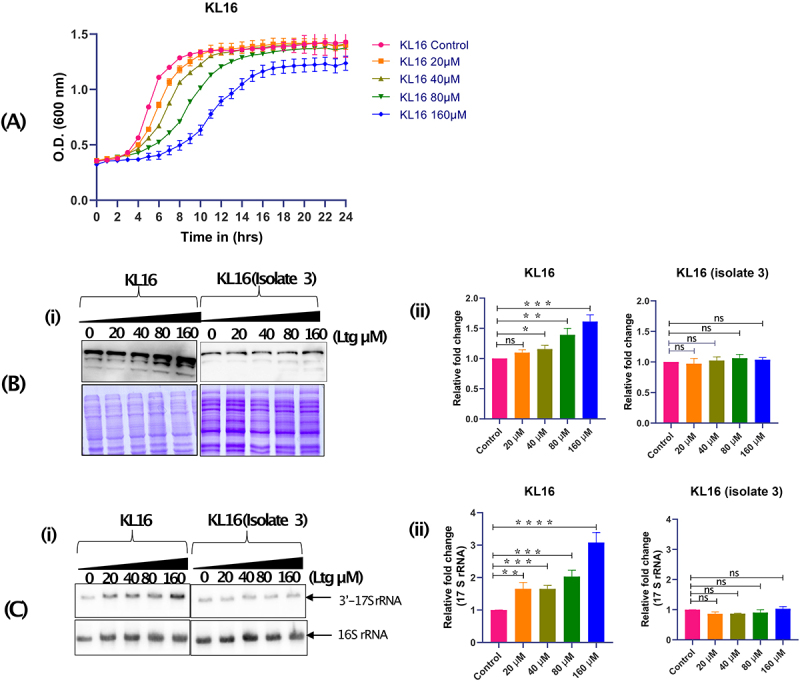
(A) Growth curves showing effect of Ltg in KL16 having IF2_WT_ in presence of increasing amount of Ltg (20, 40, 80 and 160 µM). The line joins the mean of three replicates, and error bars represent the standard deviation. (B) Immunoblot showing cytoplasmic level of IF2 in KL16 and KL16 isolate 3 (Ltg^R^) in the absence and presence of (20, 40, 80 and 160 µM) Ltg. Note- in western blot three different band represents 3 isoforms of IF2 (i.e. α, β and γ) (i). Quantitation of IF2 in KL16 and KL16 isolate 3 under control and treated condition (ii). Data are shown as mean ± SD of three independent experiments. Statistical significance *p < 0.05. (C) Northern blot showing level of immature (17S) and mature (16S) rRNAs in KL16 and KL16 isolate 3 in the absence and presence of Ltg (20, 40, 80 and 160 µM) (i). Quantitation of immature 16S rRNA in KL16 and KL16 isolate 3 under control and treated condition (ii). Data are shown as mean ± SD of three independent experiments. Statistical significance ***P < 0.001.

To investigate the effects of Ltg, we checked the growth of KL16 in LB broth in the presence of increasing drug concentrations (0, 20, 40, 80, 160 µM) at 28°C. As shown in Figure 4A, the growth of KL16 was inhibited linearly in the presence of the increasing amount of Ltg. As Ltg binds IF2, we checked for any alterations in the levels of cellular IF2 with the increasing amount of Ltg. Immunoblot analysis with anti-IF2 antibodies showed that treatment with Ltg resulted in an increase in IF2_WT_ levels in KL16 (Figure 4B, panels i and ii). However, the IF2 levels remained unaffected in the Ltg^R^ isolate (Figure 4B, panels i and ii). The Coomassie blue stained gel panels below the immunoblots (Figure 4B, panel i and ii) show that for the analysis in each strain, equal amounts of total proteins were used. Also, the Ltg treatment resulted in a slight increase in 17S rRNA in KL16 but not in the Ltg^R^ isolate (Figure 4C, panels i and ii).

### Lamotrigine does not affect the ribosome dependent GTPase activity of IF2, and dipeptide formation

**Figure 5.**
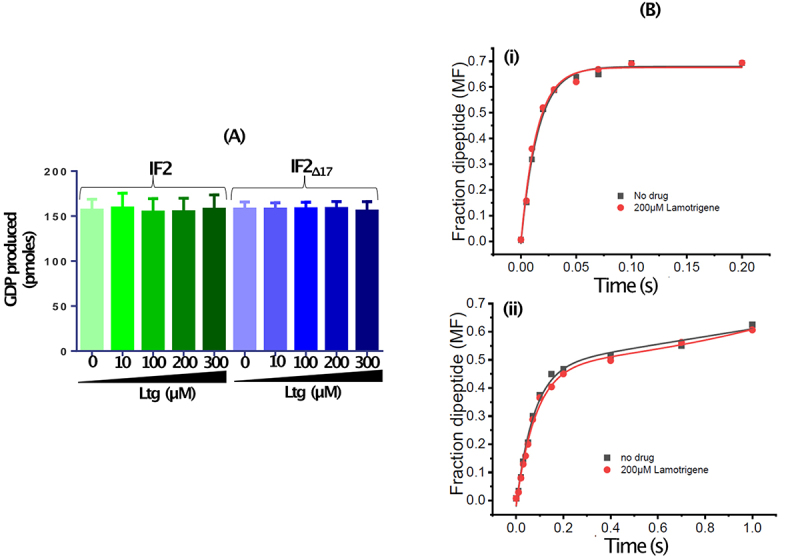
(A) *In vitro* GTPase activity of IF2_WT_ and IF2_Δ17_ in the presence of increasing amount of Ltg. (B). Time course of f[3 H] Met-Phe dipeptide formation in quench-flow starting with (i) preformed 70S IC (ii) 30S pre-IC, in the presence (red circles) and in absence (black squares) of Ltg (200 µM).

The earlier study [17] showed that Ltg did not have any impact on *in vitro* protein synthesis. As our *in vivo* experiments show that Ltg impacts IF2_WT_ mediated role of i-tRNA, we investigated the effects of Ltg on IF2 function. The presence of Ltg (10 µM to 300 µM), even at its highest concentration (300 µM), did not alter the ribosome-dependent GTPase activity of IF2_WT_ or IF2_Δ17_ (Figure 5A and S5). Likewise, when we determined the rate of productive initiation by dipeptide formation in a single turnover fast kinetics assay, starting with either the preformed 70S initiation complex (70S-IC) (Figure 5B, i) or the ribosomal subunits in the form of 30S pre-IC and 50S (Figure 5B, ii), untreated and Ltg (200 µM) treated reactions were indistinguishable.

### Lamotrigine treatment allows initiation with elongator tRNA

**Figure 6.**
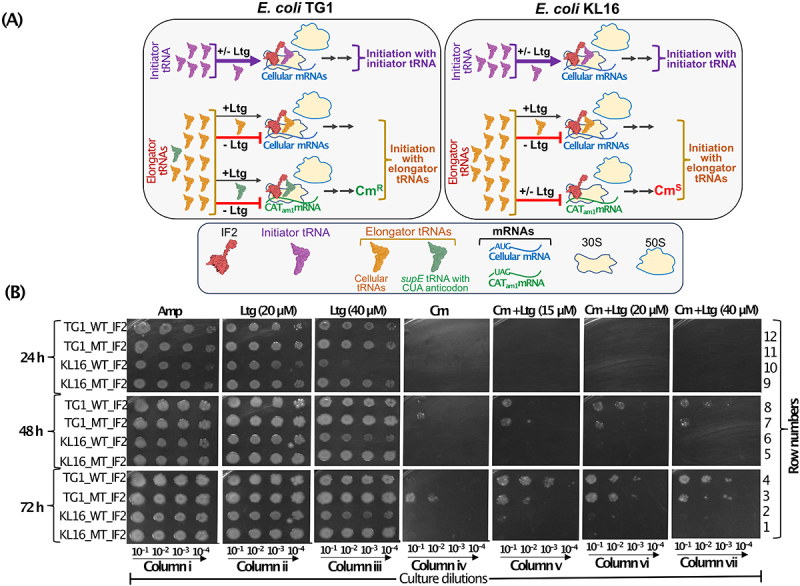
(A) Schematic showing that under normal condition, IF2 has high specificity for i-tRNA and under Ltg treated condition the high specificity of IF2 is breached to now use even the elongator tRNAs (including *supE* in the TG1 strain). The schematic shows how use of *supE* serves as a reporter for the use of elongator tRNAs in initiation in the CAT_am1_ based reporter mRNA (where the start codon AUG is replaced with UAG). Use of *supE* elongator tRNA (having CUA anticodon) present in *E. coli* TG1 (left side box) confers chloramphenicol resistance (Cm^R^) in the presence of Ltg. However, the *E. coli* KL16 which lacks such a tRNA remains chloramphenicol sensitive (Cm^S^) in spite of harbouring the CAT_am1_ reporter mRNA. (B) plate dilution assays showing growth of *E. coli* TG1 and *E. coli* KL16 having IF2_WT_ (shown as WT_IF2) and IF2_Δ17_ (shown as MT_IF2) in presence of Amp only (column i), in presence of 20 µM and 40 µM Ltg only (columns ii and iii), in presence of Cm only (column iv), in presence of Cm + increasing concentrations (15, 20 and 40 µM) of Ltg (columns v, vi and vii). Growth was captured at 24, 48 and 72 h.

As the ribosome-dependent GTPase activity of IF2 and its activity in dipeptide formation remain unaffected in the presence of Ltg (Figure 5), we asked if Ltg acts by altering the fidelity of IF2 function. Does it allow initiation with elongator tRNA? To test this, we used a plasmid-based reporter of chloramphenicol acetyltransferase, pCAT_am1,_ wherein the start codon, AUG, was converted to UAG [40]. For this experiment (Figure 6A,B), we used *E. coli* TG1 harbouring an elongator tRNA having CUA anticodon (suppressor tRNA, *supE*). It may be noted that since in the TG1 strain, there are no initiator tRNAs having CUA anticodon, in this assay system, any initiation (measured as Cm^R^) from the UAG initiation codon of the CAT_am1_ reporter mRNA must occur with the *supE* elongator tRNA^Gln^ (harbouring CUA anticodon). Thus, the TG1 strain harbouring pCATam1 provided us with a system to probe for any initiation with elongator tRNAs in *E. coli*. The KL16 strain, which does not harbour any tRNAs (elongator or initiator) with CUA anticodon, was used as a negative control.

The plate dilution spotting assay shows that TG1 and KL16 strains harbouring IF2_Δ17_ (MT_IF2) as expected, grow well in the presence of Ltg as compared to strains having IF2_WT_ (WT_IF2) (Figure 6B, columns ii and iii, compare row 12 with 11; and 10 with 9, 24 h). There is complete inhibition of growth in the presence of Cm alone at 24 h of incubation (column iv, top panel). Upon keeping the plates for 48 h and 72 h, we observed growth in TG1 having IF2_Δ17_ (Figure 6B, column iv, rows 3 and 7), but not in TG1 having IF2_WT_ (Figure 6B, column iv, rows 4 and 8). No growth on Cm alone plate was seen for KL16 strains (Figure 6B, column iv, rows 1 and 2; 5 and 6). These observations suggest that IF2_Δ17_ is compromised for its fidelity to allow initiation with the *supE* elongator tRNA. Importantly, the presence of Ltg allowed even the IF2_WT_ to use the *supE* tRNA in the initiation and growth on Cm (Figure 6B, columns v, vi and vii, rows 4 and 8), suggesting that Ltg compromises the fidelity of IF2 in allowing initiation with elongator tRNAs in general. Under the same conditions, no growth on Cm was seen in KL16 that lacks *supE* (Figure 6B, column v, vi and vii, rows 2 and 6). Overall, this experiment shows that the presence of Ltg lowered the fidelity of IF2 to now allow initiation with elongator tRNAs (as suggested by initiation by *supE* from CATam1). Such a compromise in the specificity of IF2 function would also compromise the i-tRNA function in ribosome biogenesis.

### Lamotrigine treatment results in increased retention of RbfA on 30S subunit

**Figure 7.**
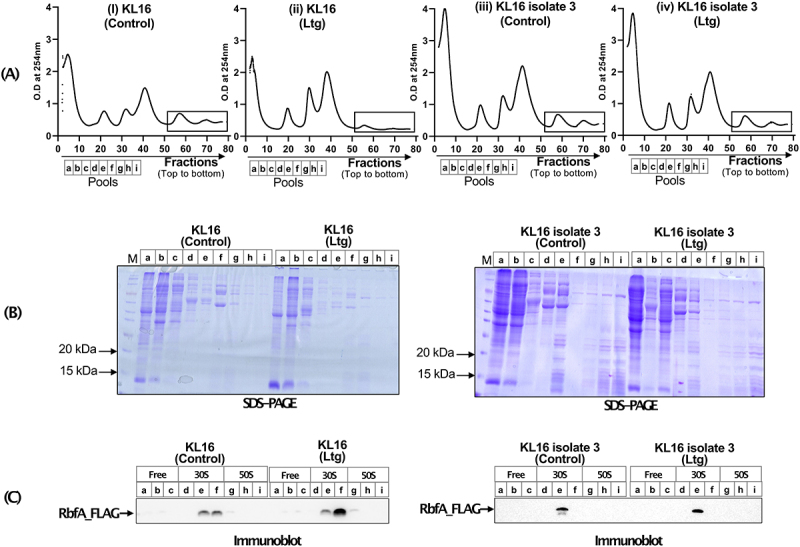
(A) Polysome profile of KL16 and KL16 isolate 3 in absence and presence of Ltg, (rectangle area showing comparative level of polysome). Pooled fractions representing ribosome free, 30S and 50S regions, used to analyse proteins from are indicated by fractions a, b, c, d, e, f and i. (B) 12% SDS-PAGE showing separation of protein from different pooled fraction (a to i). (C) Immunoblot of the gels in (B) showing the levels of FLAG_RbfA in different fractions in KL16 and KL16 isolate 3 in absence and presence of Ltg.

Further to understand Ltg toxicity, we checked for the level of RbfA on 30S subunit. To carry out this experiment, we performed polysome profiling (Figure 7A) of the KL16 *rbfA*-FLAG and KLl6 isolate 3 *rbfA*-FLAG strains (in these strains, the genomic copy of *rbfA* was tagged with FLAG-tag [11]. The polysome profiles showed that Ltg treatment decreased the abundance of polysomes in KL16 *rbfA*-FLAG (compare panels i and ii) but not in KLl6 isolate 3 *rbfA*-FLAG (compare panels iii and iv) strains. We then precipitated the total proteins from the continuous fractions corresponding to free, 30S and 50S and analysed the pooled fractions (a to i) on SDS-PAGE (Figure 7B), followed by immunoblotting with anti-FLAG antibodies (Figure 7C). As observed in Figure 7C, Ltg treatment resulted in increased levels of RbfA on the 30S subunit as compared to the Ltg untreated control (Figure 7C). In a similar experiment performed on KLl6 isolate 3 *rbfA*-FLAG strain (Ltg^R^) as a control, the RbfA levels remained unaffected under Ltg-treated condition (Figure 7C). Further, as a control, when we checked the cytoplasmic level of RbfA under Ltg untreated and treated conditions, the total RbfA levels in the two strains remained unaffected (Fig. S6). These observations suggest that the increased level of RbfA on 30S subunit under Ltg-treated conditions in KL16 *rbfA*-FLAG is due to its increased retention of RbfA on 30S rather than its increased expression. Interestingly, as Ltg imbalances IF2/i-tRNA ratio by resulting in increased levels of IF2, we noted that even under the conditions of low level overexpression of IF2 (from pACDH-IF2) or decreased i-tRNA in Δ*metZWV* strain, there is increased accumulation of RbfA on 30S (Fig. S7).

## Discussion

Stokes et. al [17] screened ~ 30000 compounds to identify molecules that conferred cold sensitive phenotype (a hallmark feature of the ribosome biogenesis defect) to *E. coli*. They identified Ltg, an anticonvulsant drug, as a small molecule that caused ribosome biogenesis defect in *E. coli*. Ltg interacted with IF2 and the mutations in its domain II (amino acid 158–290) conferred Ltg^R^. This domain is distanced from the G domain (amino acids 390 to 559) which binds GTP/GDP, and the C-terminal domain (amino acids 560 to 890) which binds i-tRNA. The sequence of the initial region of the IF2 N-terminal is variable in different bacteria, and it does not appear to be very essential for the basic translational process [19]. Also, it was shown that deletion of the first 294 amino acids encompassing both the domains I and II of the IF2 N-terminal carried out all its functions in translation initiation [41]. IF2 possesses two different sites of interaction with 30S ribosome, one is located at N-terminal region and second in the G domain. However, unlike the G-domain interaction, the N-terminal interaction is independent of the presence of GTP, IF1 or i-tRNA [41]. Thus, taken together, the lack of a direct impact of Ltg on the *in vitro* ribosome dependent GTPase activity, and the i-tRNA dependent initiation activities of IF2 in both the earlier [17] and our current fast kinetics experiments, may not be surprizing. In fact, even the rate of dipeptide formation (starting with 30S pre-IC along with fMet and the initiation factors) remains unchanged in the presence of Ltg (Figure 5). Thus, these observations clearly point to the impact of Ltg on a novel function of IF2 on the ribosome.

During the initiation step, IF2 locates i-tRNA at the P site of 30S ribosome by involving interaction with its C-terminal region (amino acids 560–890) (Figure 1A). We earlier showed that i-tRNA, at the stage of initiation complex formation during the pioneering round of initiation, plays a role in ribosome biogenesis [20] where we have shown that the binding of i-tRNA to P site on the 30S subunit facilitates processing of 3’ end of 16S rRNA, assigning an additional role to i-tRNA as a biogenesis factor. In fact, such a role of IF2 is similar to the role of its ortholog (eIF5B) in yeast, where the latter is involved in the ribosomal quality control prior to their use [42]. Thus, we investigated the effects of overexpression of IF2 and i-tRNA individually or together on the Ltg mediated effects on *E. coli* KL16. The excess IF2 causes growth defect in *E. coli*, which is enhanced with the presence of Ltg (Figure 1C, lane 2, and Figure 2A, lane 4). Ltg itself also results in increased abundance of IF2 in cell (Figure 4B). Further, the data in Figure 2B show that IF2 overexpression based toxicity is synthetic with i-tRNA deficiency. Earlier, we have shown that under i-tRNA deficient condition, the P site on 30S subunit could be occupied by elongator tRNAs [43]. These observations reveal that an optimal stoichiometry of i-tRNA and IF2 is necessary for both the normal growth of *E. coli* and in rescuing the strains from Ltg toxicity. Interestingly, imbalances in i-tRNA/IF2 stoichiometry caused by overexpression of IF2 or deletion of i-tRNA genes, also result in increased retention of RbfA on 30S subunit (Fig. S8).

Further, we show that under the conditions of excess IF2, especially in the presence of Ltg, even the elongator tRNAs may occupy the ribosomal P-site (Figure 6B) and compromise ribosome maturation (Figure 4C). In fact, we had earlier shown that the highly conserved 3GC base pairs in the anticodon stem of i-tRNA play a crucial role in ribosome maturation [20]. Consistent with our earlier observation, the 3GC mutant i-tRNA caused toxicity was synthetic with Ltg, and the toxicity increased further upon simultaneous overexpression of IF2 (Figure 2A). It may be mentioned that the 3GC mutant i-tRNA is efficiently aminoacylated and formylated [40] and binds with IF2. Thus, these genetic interactions (between i-tRNA/IF2 and Ltg) provide strong evidence that Ltg compromises the role of i-tRNA in ribosome biogenesis. Further, the CAT_am1_ based reporter assays suggest that the fidelity of IF2 is compromised in Ltg-treated cells and instead of just using i-tRNA they can now also use elongator tRNA for initiation. In addition, the sucrose density gradient profiles using *in vivo* samples indicate that there is a decrease in polysome population in KL16 upon Ltg treatment of the cultures (Figure 7A). Decreases in polysome population are often an indication of decreased rate of initiation. Thus, even though the *in vitro* experiments fail to detect the effect of Ltg in translation initiation, we argue that the compromise in the fidelity of IF2 (to allow, at least in part, binding of elongator tRNAs) decreases the rate of *in vivo* initiation from correct initiation codon, and also adversely impacts ribosome biogenesis.

The immature 30S ribosomes are characterized by the increased presence of RbfA on them [44]. The polysome profiles followed by immunoblotting for RbfA show that RbfA levels on the 30S ribosomes increases under Ltg treated conditions. These observations further suggest that Ltg compromises the biogenesis pathway by interfering with the release of RbfA from 30S or perhaps stabilizing rRNA structure that continues to retain it. The presence of excess of i-tRNA/IF2 complex may facilitate transition of the rRNA structure to the state that facilitates RbfA release. It may also be possible that presence of domain II (amino acid 158–290) allows binding of RbfA over 30S subunit and subsequent binding and/or proper positioning of i-tRNA at P site might release RbfA from 30S subunit via interaction with IF2α through C terminal region of IF2α. Finally, the present study shows that small molecules like Ltg, impacting ribosome maturation, would help exploration of the complex process of the ribosome biogenesis.

## Supplementary Material

Supplemental Material

## Data Availability

The data that support the findings of this study are available from the corresponding author, UV, upon reasonable request.

## References

[1] Ramakrishnan V, Moore PB. Atomic structures at last: the ribosome in 2000. Curr Opin Struct Biol. 2001;11(2):144–154. doi: 10.1016/S0959-440X(00)00184-6 11297922

[2] Kaczanowska M, Rydén-Aulin M. Ribosome biogenesis and the translation process in Escherichia coli. Microbiol Mol Biol Rev. 2007;71(3):477–494. doi: 10.1128/MMBR.00013-07 17804668 PMC2168646

[3] Shajani Z, Sykes MT, Williamson JR. Assembly of bacterial ribosomes. Annu Rev Biochem. 2011;80(1):501–526. doi: 10.1146/annurev-biochem-062608-160432 21529161

[4] Traub P, Nomura M. Structure and function of E. coli ribosomes. V. Reconstitution of functionally active 30S ribosomal particles from RNA and proteins. Proc Natl Acad Sci U S A. 1968;59(3):777–784. doi: 10.1073/pnas.59.3.777 4868216 PMC224743

[5] Nierhaus KH, Dohme F. Total reconstitution of 50S subunits from Escherichia Coli ribosomes. Methods Enzymol. 1979;59:443–449. 374949 10.1016/0076-6879(79)59106-x

[6] Kressler D, Hurt E, Baßler J. Driving ribosome assembly. Biochim Biophys Acta Mol Cell Res. 2010;1803(6):673–683. doi: 10.1016/j.bbamcr.2009.10.009 19879902

[7] Datta PP, Wilson DN, Kawazoe M, et al. Structural aspects of RbfA action during small ribosomal subunit assembly. Mol Cell. 2007;28(3):434–445. doi: 10.1016/j.molcel.2007.08.026 17996707 PMC2118056

[8] Guo Q, Yuan Y, Xu Y, et al. Structural basis for the function of a small GTPase RsgA on the 30S ribosomal subunit maturation revealed by cryoelectron microscopy. Proc Natl Acad Sci U S A. 2011;108(32):13100–13105. doi: 10.1073/pnas.1104645108 21788480 PMC3156173

[9] Goto S, Kato S, Kimura T, et al. RsgA releases RbfA from 30S ribosome during a late stage of ribosome biosynthesis. EMBO J [Internet]. 2011;30(1):104–114. doi: 10.1038/emboj.2010.291 21102555 PMC3020115

[10] Sharma IM, Woodson SA. RbfA and IF3 couple ribosome biogenesis and translation initiation to increase stress tolerance. Nucleic Acids Res. 2020;48:359–372. doi: 10.1093/nar/gkz1065 31728529 PMC7145577

[11] Lahry K, Gopal A, Kumar Sahu A, et al. An alternative role of RluD in the fidelity of translation initiation in Escherichia coli. J Mol Biol [Internet]. 2022;434(12):167588. doi: 10.1016/j.jmb.2022.167588 35439479

[12] Roy-Chaudhuri B, Kirthi N, Culver GM. Appropriate maturation and folding of 16S rRNA during 30S subunit biogenesis are critical for translational fidelity. Proc Natl Acad Sci U S A. 2010;107(10):4567–4572. doi: 10.1073/pnas.0912305107 20176963 PMC2842029

[13] Phunpruch S, Warit S, Suksamran R, et al. A role for 16S rRNA dimethyltransferase (ksgA) in intrinsic clarithromycin resistance in Mycobacterium tuberculosis. Int J Antimicrob Agents [Internet]. 2013;41(6):548–551. doi: 10.1016/j.ijantimicag.2013.02.011 23541074

[14] Ilina EN, Malakhova MV, Bodoev IN, et al. Mutation in ribosomal protein S5 leads to spectinomycin resistance in Neisseria gonorrhoeae. Front Microbiol. 2013;4:1–7. doi: 10.3389/fmicb.2013.00186 23847609 PMC3706878

[15] Ellis SR. Nucleolar stress in diamond blackfan anemia pathophysiology. Biochim Biophys Acta - Mol Basis Dis [Internet]. 2014;1842(6):765–768. doi: 10.1016/j.bbadis.2013.12.013 24412987

[16] Shi Z, Barna M. Translating the genome in time and space: specialized ribosomes, RNA regulons, and RNA-Binding proteins. Annu Rev Cell Dev Biol. 2015;31(1):31–54. doi: 10.1146/annurev-cellbio-100814-125346 26443190

[17] Stokes JM, Davis JH, Mangat CS, et al. Discovery of a small molecule that inhibits bacterial ribosome biogenesis. Elife. 2014;3:e03574. doi: 10.7554/eLife.03574 25233066 PMC4371806

[18] Sacerdot C, Vachon G, Laalami S, et al. Both forms of translational initiation factor IF2 (α and β) are required for maximal growth of Escherichia coli. Evidence for two translational initiation codons for IF2β. J Mol Biol. 1992;225(1):67–80. doi: 10.1016/0022-2836(92)91026-L 1374802

[19] Gualerzi CO, Pon CL. Initiation of mRNA translation in bacteria: structural and dynamic aspects. Cell Mol Life Sci. 2015;72(22):4341–4367. doi: 10.1007/s00018-015-2010-3 26259514 PMC4611024

[20] Shetty S, Varshney U. An evolutionarily conserved element in initiator tRNAs prompts ultimate steps in ribosome maturation. Proc Natl Acad Sci U S A. 2016;113(41):E6126–34. doi: 10.1073/pnas.1609550113 27698115 PMC5068261

[21] Milon P, Carotti M, Konevega AL, et al. The ribosome-bound initiation factor 2 recruits initiator tRNA to the 30S initiation complex. EMBO Rep [Internet]. 2010;11(4):312–316. doi: 10.1038/embor.2010.12 20224578 PMC2854590

[22] Low B. Formation of merodiploids in matings with a class of Rec- recipient strains of Escherichia coli K12. Proc Natl Acad Sci U S A. 1968;60(1):160–167. doi: 10.1073/pnas.60.1.160 4873517 PMC539096

[23] Sambrook J, Fritsch ER, Maniatis T. Molecular cloning: a laboratory manual. 2nd ed. Cold Spring Harbor, NY: Cold Spring Harbor Laboratory Press; 1989.

[24] Studier FW, Moffatt BA. Use of bacteriophage T7 RNA polymerase to direct selective high-level expression of cloned genes. J Mol Biol. 1986;189(1):113–130. doi: 10.1016/0022-2836(86)90385-2 3537305

[25] Rao AR, Varshney U. Characterization of Mycobacterium tuberculosis ribosome recycling factor (RRF) and a mutant lacking six amino acids from the C-terminal end reveals that the C-terminal residues are important for its occupancy on the ribosome. Microbiology. 2002;148(12):3913–3920. doi: 10.1099/00221287-148-12-3913 12480895

[26] Rahul G, Grasso D, Datta PP, et al. A single mammalian mitochondrial translation initiation factor functionally replaces two Bacterial factors. Molecular Cell. 2008;29(2):180–190. doi: 10.1016/j.molcel.2007.11.021 18243113 PMC2605297

[27] Samhita L, Virumäe K, Remme J, et al. Initiation with elongator tRNAs. J Bacteriol. 2013;195(18):4202–4209. doi: 10.1128/JB.00637-13 23852868 PMC3754738

[28] Varshney U, RajBhandary UL. Initiation of protein synthesis from a termination codon. Proc Natl Acad Sci U S A. 1990;87(4):1586–1590. doi: 10.1073/pnas.87.4.1586 2406724 PMC53520

[29] Samhita L, Shetty S, Varshney U. Unconventional initiator tRNAs sustain Escherichia coli. Proc Natl Acad Sci U S A. 2012;109(32):13058–13063. doi: 10.1073/pnas.1207868109 22829667 PMC3420168

[30] Govindan A, Ayyub SA, Varshney U. Sustenance of Escherichia coli on a single tRNA^Met^. Nucleic Acids Res. 2018;46:11566–11574. doi: 10.1093/nar/gky859 30256973 PMC6265465

[31] Datsenko KA, Wanner BL. One-step inactivation of chromosomal genes in Escherichia coli K-12 using PCR products. Proc Natl Acad Sci U S A. 2000;97(12):6640–6645. doi: 10.1073/pnas.120163297 10829079 PMC18686

[32] Uzzau S, Figueroa-Bossi N, Rubino S, et al. Epitope tagging of chromosomal genes in Salmonella. Proc Natl Acad Sci U S A. 2001;98(26):15264–15269. doi: 10.1073/pnas.261348198 11742086 PMC65018

[33] Mehta P, Woo P, Venkataraman K, et al. Ribosome purification approaches for studying interactions of regulatory proteins and rnas with the ribosome. Methods Mol Biol. 2012;905:273–289. 22736011 10.1007/978-1-61779-949-5_18 PMC4607317

[34] Terasaki M, Suzuki T, Hanada T, et al. Functional compatibility of elongation factors between mammalian mitochondrial and bacterial ribosomes: characterization of GTPase activity and translation elongation by hybrid ribosomes bearing heterologous L7/12 proteins. J Mol Biol. 2004;336(2):331–342. doi: 10.1016/j.jmb.2003.12.034 14757048

[35] Kapoor I, Varshney U. Diverse roles of nucleoside diphosphate kinase in genome stability and growth fitness. Curr Genet [Internet]. 2020;66(4):671–682. doi: 10.1007/s00294-020-01073-z 32249353

[36] Mandava CS, Peisker K, Ederth J, et al. Bacterial ribosome requires multiple L12 dimers for efficient initiation and elongation of protein synthesis involving IF2 and EF-G. Nucleic Acids Res. 2012;40(5):2054–2064. doi: 10.1093/nar/gkr1031 22102582 PMC3299993

[37] Parajuli NP, Mandava CS, Pavlov MY, et al. Mechanistic insights into translation inhibition by aminoglycoside antibiotic arbekacin. Nucleic Acids Res. 2021;49(12):6880–6892. doi: 10.1093/nar/gkab495 34125898 PMC8266624

[38] Tobin C, Mandava CS, Ehrenberg M, et al. Ribosomes lacking protein S20 are defective in mRNA binding and subunit association. J Mol Biol [Internet]. 2010;397(3):767–776. doi: 10.1016/j.jmb.2010.02.004 20149799

[39] Shetty S, Nadimpalli H, Shah RA, et al. An extended shine–dalgarno sequence in mRNA functionally bypasses a vital defect in initiator tRNA. Proc Natl Acad Sci U S A. 2014;111(40):E4224–33. doi: 10.1073/pnas.1411637111 25246575 PMC4210040

[40] Das G, Thotala DK, Kapoor S, et al. Role of 16S ribosomal RNA methylations in translation initiation in Escherichia coli. EMBO J. 2008;27(6):840–851. doi: 10.1038/emboj.2008.20 18288206 PMC2274930

[41] Caserta E, Tomšic J, Spurio R, et al. Translation initiation factor IF2 interacts with the 30 S ribosomal subunit via two separate binding sites. J Mol Biol. 2006;362(4):787–799. doi: 10.1016/j.jmb.2006.07.043 16935296

[42] Strunk BS, Novak MN, Young CL, et al. Joining of 60S subunits and a translation-like cycle in 40S ribosome maturation. 2012;150: 111–121. doi: 10.1016/j.cell.2012.04.044 PMC3615461 22770215

[43] Kapoor S, Das G, Varshney U. Crucial contribution of the multiple copies of the initiator tRNA genes in the fidelity of tRNA^fMet^ selection on the ribosomal P-site in Escherichia coli. Nucleic Acids Res. 2011;39(1):202–212. doi: 10.1093/nar/gkq760 20798174 PMC3017606

[44] Maksimova EM, Korepanov AP, Kravchenko OV, et al. RbfA is involved in two important stages of 30S subunit assembly: formation of the central pseudoknot and docking of helix 44 to the decoding center. Int J Mol Sci. 2021;22(11):22. doi: 10.3390/ijms22116140 PMC8201178 34200244

