# Supplementary material for: Lamotrigine compromises the fidelity of initiator tRNA recruitment to the ribosomal P-site by IF2 and the RbfA release from 30S ribosomes in Escherichia coli
Source: RNA Biol. 2023 Sep 7;20(1):681–92. doi: 10.1080/15476286.2023.2253395 (PMC10486304; doi:10.1080/15476286.2023.2253395)
Supplement: Supplemental Material [file KRNB_A_2253395_SM8337.pdf]

Fig S1

(A)

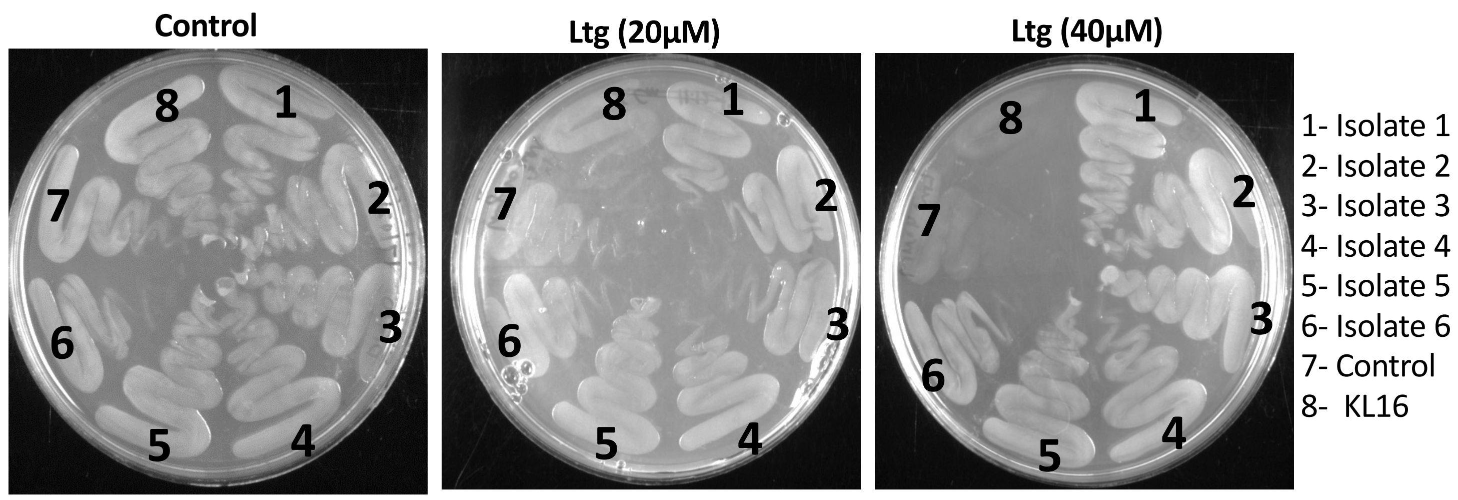

(B)

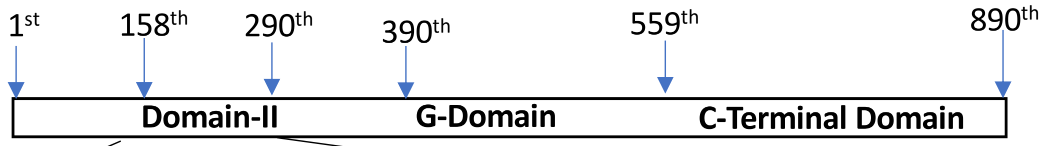


```
Supp_1     GCAGAGCTCAAGCGTAAAGCTGAAGAAGAAGCG----------TTGAAGAAGAAGCGCGT     590
Supp_5     GCACAGCTCAAGCGTTAAGCTGAAGAAGAAGCGCGTCGTAAACTCGAAGAAGAAGCGCGT     600
Supp_6     GCAGAGCTCAAGCGTAAAGCTGAAGAAGAA------------------------------     570
Supp_3     GAAGCACGTCGCG-----------------------------------------------     553
ECO        GCAGAGCTCAAGCGTAAAGCTGAAGAAGAAGCGCGTCG----------------------     578
Supp_2     GCAGAGCTCAAGCGTAAAGCTGAAGAAGAAGCGCGTCG----------------------     578
Supp_4     GCAGAGCTCAAGCGTAAAGCTGAAGAAGAAGC----------------------------     572
           * *   *

Supp_1     CGTAAACTCGAAGAAGAAGCACGTCGCGTTGCTGAAGAAGCACGTCGTATGGCGGAAGAA     650
Supp_5    CGTAAACTCGAAGAAGAAGCACGTCGCGTTGCTGAAGAAGCACGTCGTATGGCGGAAGAA      660
Supp_6     ------------------GCGCGTCGCGTTGCTGAAGAAGCACGTTGTATGGCGGAAGAA     612
Supp_3     ----------TTGCTGAAGAAGCACGTCGTATGGCGGAAGCACGTCGTATGGCGGAAGAA     603
ECO        --TAAACTCGAAGAAGAAGCACGTCGCGTTGCTGAAGAAGCACGTCGTATGGCGGAAGAA     636
Supp_2     --TAAACTCGAAG---AAGCACGTCGCGTTGCTGAAGAAGCACGTCGTATGGCGGAAGAA     633
Supp_4     --------------------GCGTCGCGTTGCTGAAGAAGCACGTCGTATGGCGGAAGAA     612
                                    **   *   *   ********* **************
```

Fig. S1: (A) Ltg Resistant colonies (isolates 1 to 6) were isolated (Materials and Methods) and confirmed by streaking overnight grown cultures on LB agar plate without or with Ltg (20 and 40 µM) and incubation at 22°C for 24 h. **(B)** Schematic showing different region of IF2 from N to C terminal of protein IF2. The domain II is in the N- terminal region between amino acid 158 to 290. The alignment of IF2 sequences from the different isolates and the parent strain is shown. Alignment shows the deletions of few to several residues in domain II.

Fig S2

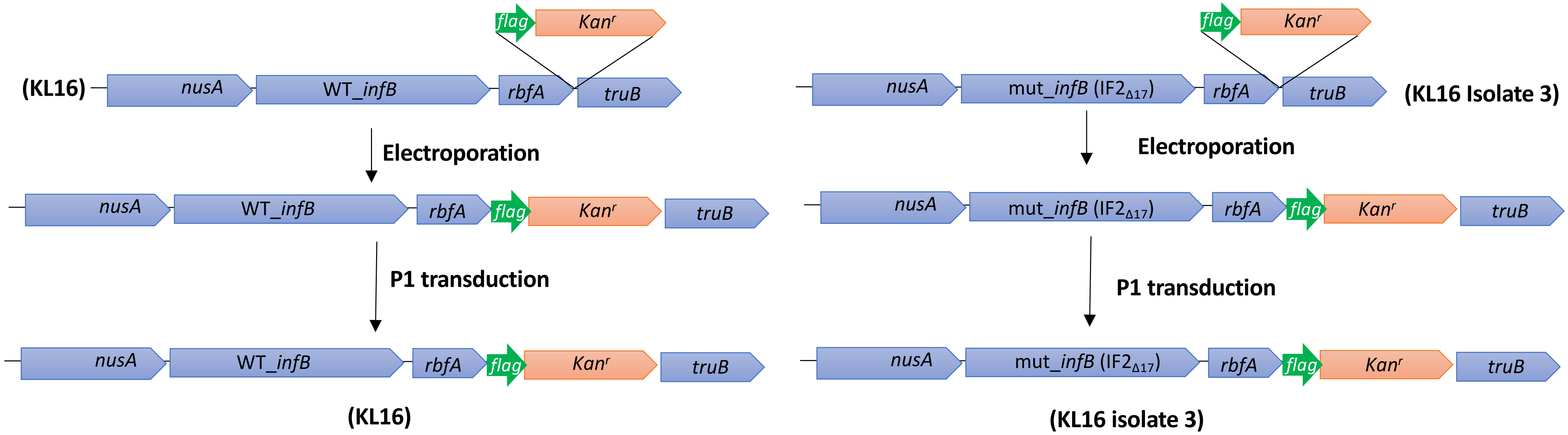

Fig. S2: Schematic showing generation of isogenic strains of *E. coli* KL16 where IF2 (*infB*) loci were tagged by insertion of *flag*-*kan* cassette downstream to *rbfA* gene. The derivatives of KL16 and KL16 isolate 3 strains were obtained by electroporation of the PCR amplicons. For further transfers of $IF2_{WT}$ and $IF2_{\Delta17}$ into fresh backgrounds of KL16, P1 transductions were used.

Fig S3

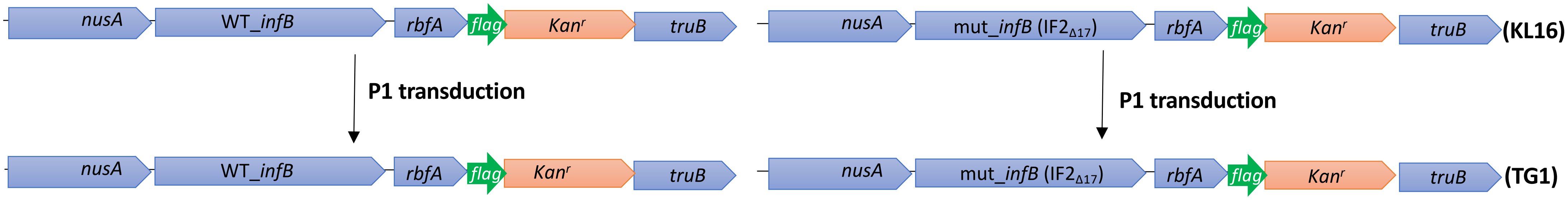

Fig. S3: Schematic showing transfer of $IF2_{WT}$ and $IF2_{\Delta17}$ from KL16 (tagged with flag-*kan*) to *E. coli* TG1 for generation of isogenic strain of *E. coli* KL16 and *E. coli* TG1 harbouring $IF2_{WT}$ and $IF2_{\Delta17}$.

Fig. S4

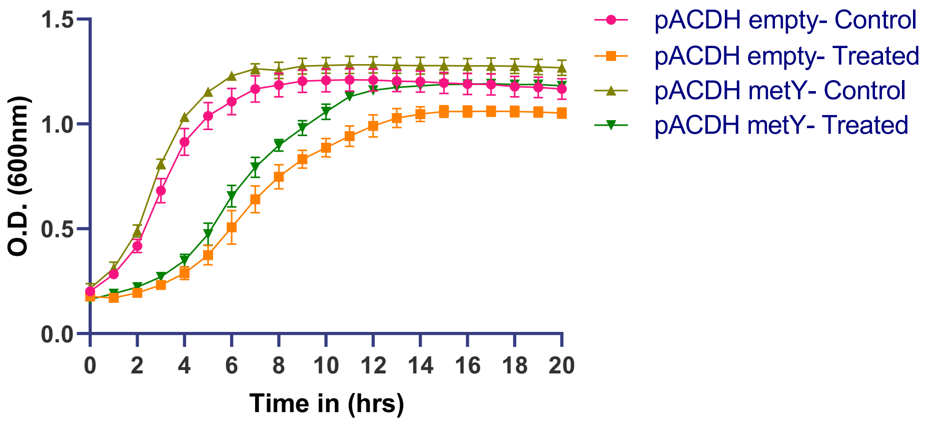

Fig. S4: Growth of KL16 having overexpressed *metY* (i-tRNA) cloned into pACDH vector in absence and presence of 40μM Ltg at 28°C

Fig S5

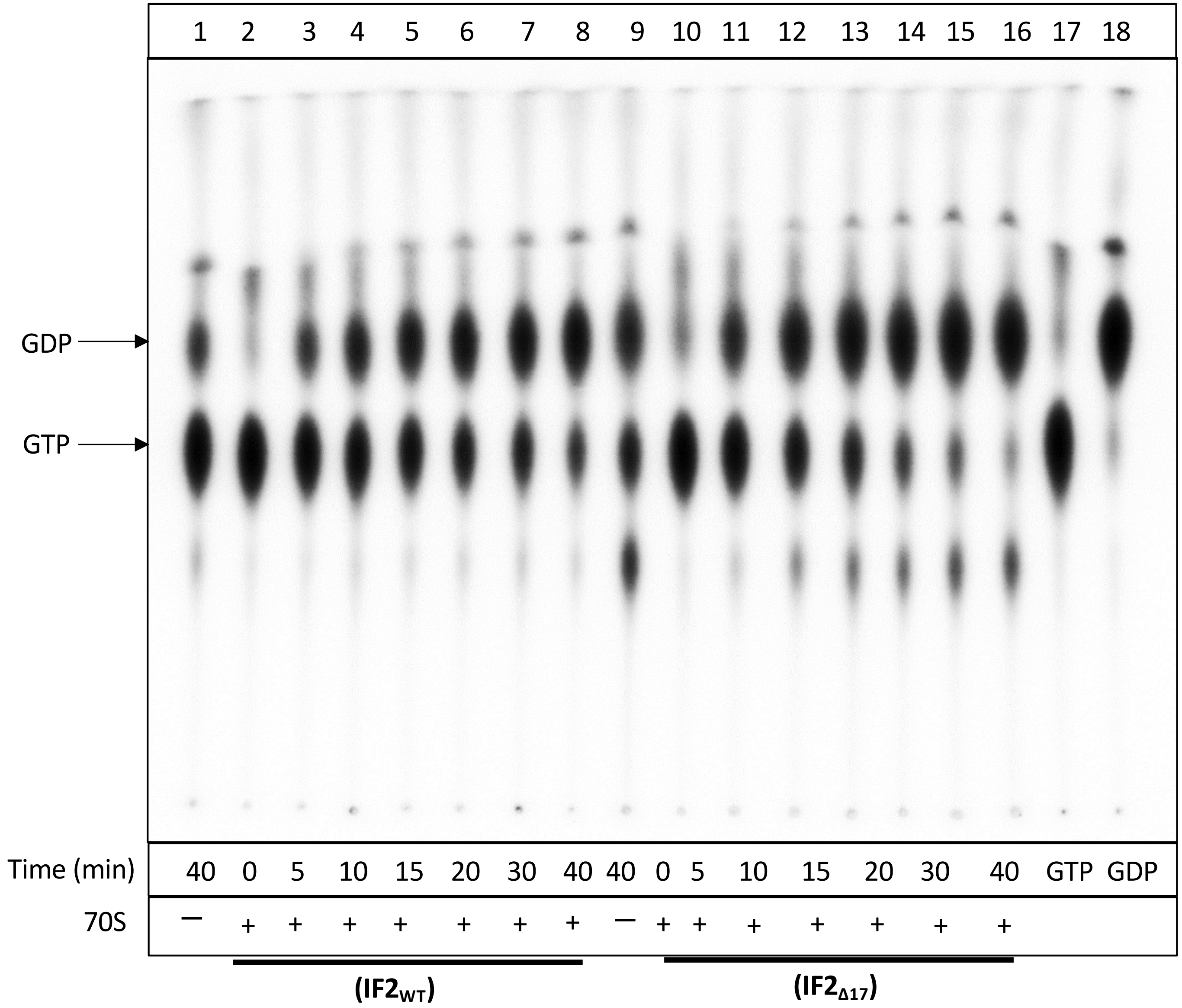

Fig. S5: TLC plate showing separation of ^{32}P (α-position) labelled GTP and GDP in reactions with IF2$_{WT}$ or IF2$_{\Delta17}$; without 70S (lanes 1 and 0), and with purified 70S for different time interval 0, 5, 10, 20, 30 and 40 min (lane 2 to 8, and 10-16, respectively). Positive control labelled GTP only (lane 17) and GDP (lane 18, generated by treatment with nucleoside diphosphate kinase, NDK).

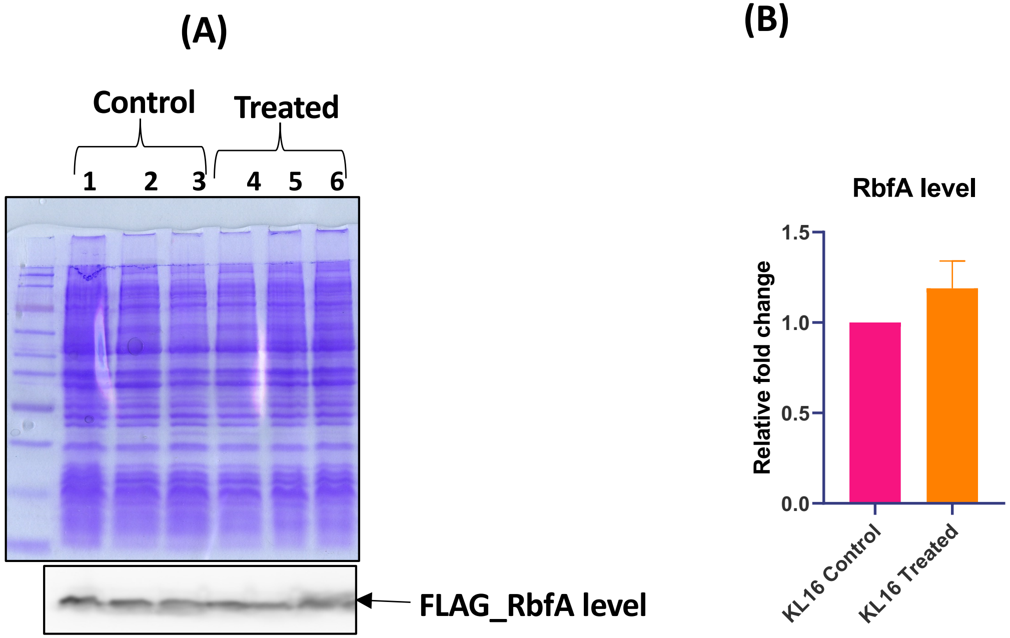

Fig. S6: (A) 12% SDS-PAGE showing separation of total cytoplasmic protein from KL16 under control (Lane 1, 2 and 3) and treated (Lane 4, 5 and 6) condition and corresponding immunoblot for RbfA-FLAG and **(B)** Quantification of normalized level of RbfA-FLAG.

Fig. S7

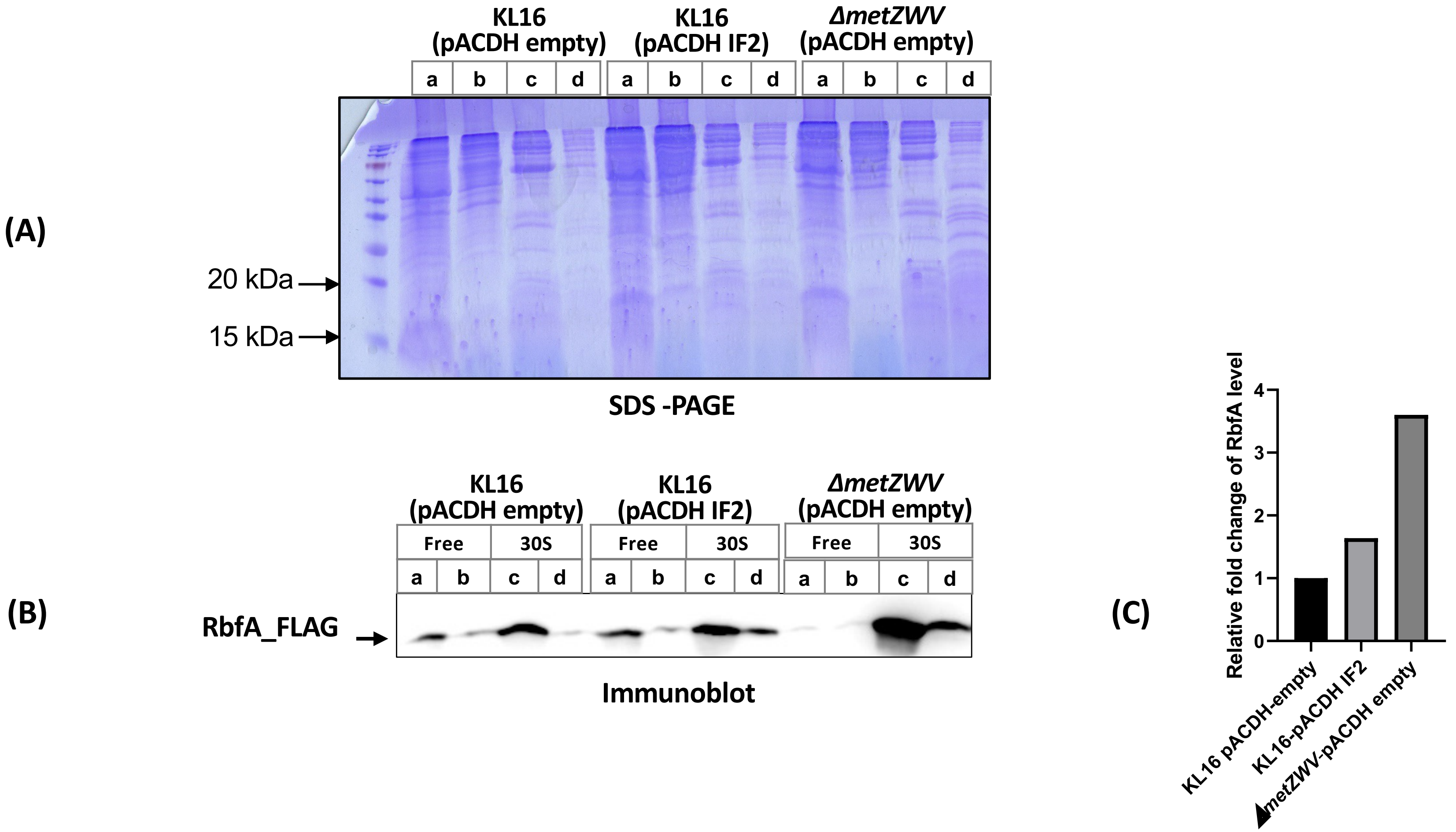

Fig. S7: Increased accumulation of RbfA in 30S with imbalances in i-tRNA/IF2 levels in *E. coli*. (A) 12% SDS-PAGE showing separation of protein from different pooled fractions of polysome profile (a, b, c and d) as a loading control. Fractions a, and b, correspond to the ribosome free region where is the fractions c, and d, correspond to the 30S ribosome. **(B)** Immunoblot of the gels shown in **(A)** showing level of RbfA in different fraction of KL16 having pACDH empty (control), pACDH-IF2 (for expression of IF2) and *ΔmetZWV* having pACDH empty vector. Quantification of the RbfA (total in fractions c, and d) in KL16 (pACDH), KL16 (pACDH-IF2) and KL16 (Δ*metZWV*) detected in **(B)** is shown in panel **(C)**.
